# Oral administration of taheebo (*Tabebuia avellanedae* Lorentz ex Griseb.) water extract prevents DSS-induced colitis in mice by up-regulating type II T helper immune responses

**DOI:** 10.1186/s12906-017-1952-4

**Published:** 2017-09-06

**Authors:** Hyun Jung Park, Sung Won Lee, Dong-Joo Kwon, Seong-Il Heo, Se-Ho Park, Sun Young Kim, Seokmann Hong

**Affiliations:** 1 Department of Integrative Bioscience and Biotechnology, Institute of Anticancer Medicine Development, Sejong University, Seoul, 143-747 South Korea; 2 School of Life Sciences and Biotechnology, Korea University, Seoul, 136-701 South Korea; 3 Hongcheon Institute of Medicinal Herb, Hongcheon, Kangwon-Do 250-930 South Korea

**Keywords:** Taheebo water extract, Dextran sulfate sodium, Intestinal inflammation, M2 macrophage, Treg cells, Th2 cells

## Abstract

**Background:**

Inflammatory bowel diseases (IBDs) are chronic inflammatory disorders that are mediated by pathogenic Th1 and Th17 cells. Previous studies have demonstrated that taheebo water extract (TWE) derived from *Tabebuia avellanedae* Lorentz ex Griseb*.,* as folk remedy, has been used to treat various inflammatory diseases. Although TWE has been previously shown to display anti-inflammatory activities, the in vivo effects of TWE on mucosal immune responses remain unclear.

**Methods:**

We examined the anti-inflammatory effects of TWE on innate immune cells such as dendritic cells (DCs) and macrophages and also on the differentiation of T helper cells. Lastly, adopting a method for dextran sulfate sodium (DSS)-induced colitis, we investigated whether the oral administration of TWE can modulate mucosal inflammatory responses.

**Results:**

We found that TWE could activate DCs to produce immunosuppressive IL10 and polarize macrophages toward an anti-inflammatory phenotype in the mesenteric lymph node (MLN). Such alterations in DCs and macrophages resulted in a significant increase in anti-inflammatory Th2 and Foxp3^+^ Treg cells and a dramatic decrease in pro-inflammatory Th1 and Th17 cells in the MLN. Upon induction of colitis with DSS treatment, TWE significantly reduced the clinical symptoms, including body weight loss and colonic tissue inflammation, by up-regulating type II T helper immune responses.

**Conclusions:**

Taken together, these data suggest that TWE is an excellent natural product with therapeutic effects to help improve inflammatory disorders such as colitis.

## Background

Inflammatory bowel diseases (IBDs) are chronic inflammatory disorders that are elicited by the breakdown of immune tolerance. IBDs are generally divided into two primary forms: ulcerative colitis (UC), which is mediated by a T helper type (Th)2-dominant condition, and Crohn’s disease (CD), which is mediated by a Th1-dominant condition [1]. Animal models for IBD have been established using dextran sulfate sodium (DSS), trinitrobenzene sulfonic acid (TNBS), and the adoptive transfer of naive T cells and these animal models quite faithfully reproduced the clinical symptoms of human IBD [1]. Host immune systems profoundly contribute to maintaining intestinal homeostasis and also controlling the pathogenesis of IBD. For example, Th17 cells are critically involved in the pathogenesis of colitis, whereas regulatory T (Treg) cells play central roles in the protection against colitis [2]. Furthermore, intestinal dendritic cells (DCs) can induce the differentiation of Th17 cells, but when stimulated by retinoic acid (RA) DCs can induce Treg cells [3]. The differentiation of intestinal forkhead box P3 (Foxp3)^+^ Treg cells is induced by RA-producing DCs, and consequently these Treg cells produce the anti-inflammatory cytokine interleukin 10 (IL10) for gut homeostasis [4]. In colitis, M1 macrophages that are polarized by interferon γ (IFNγ) or lipopolysaccharide (LPS) are associated with inflammation and gut destruction, whereas M2 macrophages that are polarized by IL4 have anti-inflammatory functions that are associated with tissue repair, which indicates that the modulation of macrophage polarization could be a therapeutic strategy for IBD [5].

*Tabebuia avellanedae* Lorentz ex Griseb., a typical tree of the Brazilian savannah, is indigenous to South America from Brazil to northern Argentina, and the products from this tree are named “ipe-roxo”, “lapacho” or “taheebo” [6]. Among these names, “taheebo” is most widely used, so we use this term hereafter. These products were used as traditional folk remedies for treating a variety of diseases [7]. Moreover, these products have been reported to possess various functions, including anti-inflammatory [8, 9], anti-bacterial [10], and anti-ulcer [11]. However, the effects of taheebo water extract (TWE) on mucosal immune responses have not been explored. Thus, in this study, we investigated whether oral administration of TWE can modulate intestinal inflammatory immune responses by employing DSS-induced murine colitis model.

## Methods

### Mice and reagents

C57BL/6 (B6) wild type (WT) mice were purchased from Jung Ang Lab Animal, Inc. (Seoul, Korea). The IL4/GFP (4get) and IFNγ/YFP (yeti) cytokine reporter mice were kindly provided by Dr. R. Locksley (University of California at San Francisco, CA, USA). Foxp3/GFP reporter B6 mice and CD11c-diphtheria toxin receptor (DTR) transgenic (tg) mice were obtained from Dr. Rho H. Seong and Dr. E. Choi, respectively (Seoul National University, Seoul, Korea). All of the mice were on a B6 genetic background, were maintained at Sejong University, and were used for experiments at 6–12 weeks of age. They were maintained on a 12-h light/12-h dark cycle in a temperature-controlled barrier facility with free access to food and water. These mice were fed with a γ-irradiated sterile diet and autoclaved tap water. In this study, age- and sex-matched mice were used for all the experiments. The animal experiments were approved by the Institutional Animal Care and Use Committee at Sejong University (SJ-20130802). Our experiments were conducted in a blinded and randomized trial. For the induction of colitis, DSS with a molecular weight of 36–50 kDa was purchased from MP Biomedicals (Solon, OH, USA). LPS derived from *Escherichia coli* (serotype 0111:B4) was purchased from Sigma-Aldrich (St. Louis, MO, USA).

### Preparation of TWE

Taheebo, the dried inner bark of *Tabebuia avellanedae* Lorentz ex Griseb., was purchased from Frontier Natural Products Co-op. (Norway, IA, USA). To prepare the TWE, five hundred grams of taheebo was stirred in 5 L cold water at 4 °C for 24 h. After filtration, the solution was reduced to 10% of the original volume with a 40 °C rotary evaporator under vacuum. The extracts were freeze-dried, ground to a fine powder, and stored at 4 °C until use.

### Standardization of TWE by HPLC

TWE was standardized with 6-O-(3,4-dimethoxybenzoyl)-ajugol and 6-O-(*p*-dimethoxybenzoyl)-ajugol known as constituents in *Tabebuia avellanedae* Lorentz ex Griseb. [12], using high-performance liquid chromatography (HPLC; SCL-10A, Shimadzu, Japan) installed with a UV-VIS detector (SPD-10A; Shimadzu, Japan). Sample filtration was performed using membrane filter (Maidstone, Kent, UK) with pore size 0.45 μm prior to injection. Sunfire C18 column (4.6 mm × 250 mm; Waters, USA) was used in reversed-phase chromatography. The temperature within the chamber was maintained at 30 °C. A 10 μl aliquot of the sample was injected onto a C18 reversed-phase column and the absorbance was detected at 260 nm. Sample concentration was calculated from a standard calibration curve obtained with 6-O-(3,4-dimethoxybenzoyl)-ajugol and 6-O-(*p*-dimethoxybenzoyl)-ajugol. 6-O-(3,4-dimethoxybenzoyl)-ajugol and 6-O-(*p*-dimethoxybenzoyl)-ajugol were found in TWE at a mean level of 12.29 mg/g and 16.25 mg/g, respectively.

### Flow Cytometry

The following monoclonal antibodies (mAbs) from BD Bioscience were used: fluorescein isothiocyanate (FITC)-, phycoerythrin (PE)-Cy7, or allophycocyanin (APC)-conjugated anti-CD3ε (clone 145-2C11); PE- or APC-conjugated anti-CD11c (clone HL3); PE-conjugated anti-cytotoxic T lymphocyte-associated protein 4 (CTLA4) (clone UC10-4F10–11); PE-Cy7-conjugated anti-CD11b (clone M1/70); APC-conjugated anti-F4/80 (clone BM8); PE-Cy7- or APC-conjugated anti-CD4 (RM4–5); APC-conjugated anti-CD25 (clone PC61); PE-conjugated anti-MHC II (clone M5/114.15.2); biotin-conjugated anti-CD86 (clone GL1); PE-conjugated anti-tumor necrosis factor α (TNFα) (clone MP6-XT22); PE-conjugated anti-IL6 (clone MP5-20F3); PE-conjugated anti-IL10 (clone JES5-16E3); PE-conjugated anti-IL12p40 (clone C15.6); and PE-conjugated anti-IFNγ (clone XMG1.2). The following mAbs were obtained from eBioscience (San Diego, USA): FITC- or PE-conjugated anti-IL4 (clone BVD6-24G2); FITC- or PE-conjugated anti-IL17A (clone eBio17B7); PE-conjugated anti-inducible nitric oxide synthase (iNOS) (clone CXNFT); and FITC- or PE-conjugated anti-Foxp3 (clone NRRF-30). The following mAb from R&D Systems was used: PE-conjugated anti-arginase-1. To perform surface staining, the cells were harvested and washed twice with 0.5% BSA-containing cold PBS (FACS buffer). To block the Fc receptors, the cells were incubated with anti-CD16/CD32 mAbs on ice for 10 min and were subsequently stained with fluorescence-labeled mAbs.

### Intracellular cytokine staining

To perform intracellular staining, splenocytes were incubated with brefeldin A, an intracellular protein transport inhibitor (10 μg/ml), in RPMI medium for 2 h at 37 °C. The cells were stained for cell surface markers, fixed with 1% paraformaldehyde, washed once with cold FACS buffer, and permeabilized with 0.5% saponin. The permeabilized cells were then stained for an additional 30 min at room temperature with the indicated mAbs (FITC-conjugated anti-IL17 or anti-IL4; PE-conjugated anti-IFNγ, anti-IL4, anti-IL10, anti-IL6, anti-IL12, anti-TNFα, anti-iNOS, or anti-arginase-1; FITC- or PE-conjugated isotype control rat IgG mAbs). Fixation and permeabilization were performed using a Foxp3 staining kit (eBioscience) with the indicated mAbs (FITC-conjugated anti-Foxp3; PE-conjugated anti-Foxp3; or FITC- or PE-conjugated isotype control rat IgG mAbs). More than 5000 cells per sample were acquired using a FACSCalibur, and the data were analyzed using the FlowJo software package (Tree Star, Ashland, OR, USA).

### Cell isolation by magnetic activated cell sorting (MACS) and culture

Splenic CD4^+^ T cells were isolated from mice using a MACS system (Miltenyi Biotec, Bergisch Gladbach, Germany), following the manufacturer’s instructions. The purity of CD4^+^ T cells was >97% after MACS. Mesenteric lymph nodes (MLNs) were aseptically removed, and single-cell suspensions of the MLN were obtained by homogenization and passing through a 70 μm nylon cell strainer. Primary macrophages and the RAW264.7 macrophage cell line were cultured in RPMI 1640 (Gibco BRL, USA) culture media supplemented with 10% FBS, 10 mM HEPES, 2 mM L-glutamine, 100 units/mL penicillin-streptomycin, and 5 mM 2-mercaptoethanol.

### In vivo depletion of DCs

In order to deplete CD11c^+^ DC populations, CD11c-DTR tg recipient mice were intraperitoneally injected with diphtheria toxin (DT) (120 ng/mouse) twice 3 days apart and subsequently mice were sacrificed for experiments 2 days after the second injection.

### Determination of cell viability

To examine the effect of TWE on cell viability of RAW264.7 cells, RAW264.7 cells were seeded in 24-well plates at a cell density of 1 × 10^6^ cells/ml (2 × 10^5^ cells/well) and stimulated with either TWE (100, 300, 900, and 2700 μg/ml) or LPS (1 μg/ml) for 12 h at 37 °C in a CO_2_ incubator. Total cells were washed in PBS, and 100 μl annexin V buffer (BD Pharmingen, San Diego, CA) containing 10 μl 7-amino actinomycin (7-AAD) was added for 15 min at room temperature in the dark. Cells were resuspended in 300 μl annexin V buffer and were analyzed within 1 h by flow cytometry. The relative cell viability (%) was expressed as a percentage (7-AAD negative) relative to the control cells.

### Polarization of macrophages with M1 or M2 stimuli

To examine the effect of TWE on the polarization towards the M2 phenotype, RAW264.7 cells (1 × 10^6^ cells) were seeded in 24-well plates and stimulated with either TWE (100, 300, and 900 μg/ml) or vehicle in the presence of IL4 (20 ng/ml) for 12 h at 37 °C in a CO_2_ incubator. In addition, to test the effect of TWE on M1 polarization, RAW264.7 cells were stimulated with either TWE (100, 300, and 900 μg/ml) or vehicle in the presence of IFNγ (100 ng/ml) for 12 h at 37 °C in a CO_2_ incubator. LPS (1 μg/ml) was used as positive control in the same culture conditions as above. After treatment, the polarization status of the RAW264.7 cells was determined by evaluating the expression of either iNOS for M1 or arginase-1 for M2 by flow cytometry.

### Induction of colonic inflammation

Before DSS treatment, two groups of B6 mice were fed with either TWE-containing or control water for 5 days. Mice in the TWE-treating group were fed with approximately 2 mg of TWE per day. Subsequently, these mice were fed with 3% (*w*/*v*) DSS in either TWE-containing or control water ad libitum for 5 days. After 5 days of DSS administration, both groups of mice were given normal control water for 5 days until sacrifice for experiments. To evaluate the clinical symptoms of DSS-induced colitis, the mice were monitored for a change in the percentage of body weight (0, none; 1, 1–10%; 2, 11–20%; 3, >20%), stool consistency (0, normal; 1, loose stool; 2, diarrhea), and bleeding (0, normal; 1, hemoccult positive; 2, gross bleeding) on a daily basis during colitis induction for 10 days. The body weight was expressed as a percentage of weight change for each individual mouse and was calculated relative to the starting body weight on day 0. These data were used to calculate a disease activity index (DAI).

### Histology

Distal colonic sections were fixed in 4% paraformaldehyde, embedded in paraffin, and cut into 6 μm sections using a microtome (RM 2235, Leica, Germany). The sections were then stained with H&E for the analysis of histological changes. The histological score of each individual mouse was measured as follows: epithelial damage (E), 0 = none; 1 = minimal loss of goblet cells; 2 = extensive loss of goblet cells; 3 = minimal loss of crypts and extensive loss of goblet cells; 4 = extensive loss of crypts; and infiltration (I), 0 = no infiltrate; 1 = infiltrate around the crypt basis; 2 = infiltrate reaching the muscularis mucosa; 3 = extensive infiltration reaching the muscularis mucosa and thickening of the mucosa with abundant edema; 4 = infiltration of the submucosa. The total histological score was calculated as E + I.

### Statistical analysis

Statistical significance was determined using the Excel software (Microsoft, USA). To compare two groups, Student’s t-test was performed. *P < 0.05, **P < 0.01, and ***P < 0.001 were considered significant.

## Results

### TWE induces an increase in maturation and production of anti-inflammatory cytokine but does not alter pro-inflammatory cytokine production in DCs

**Fig. 1:**
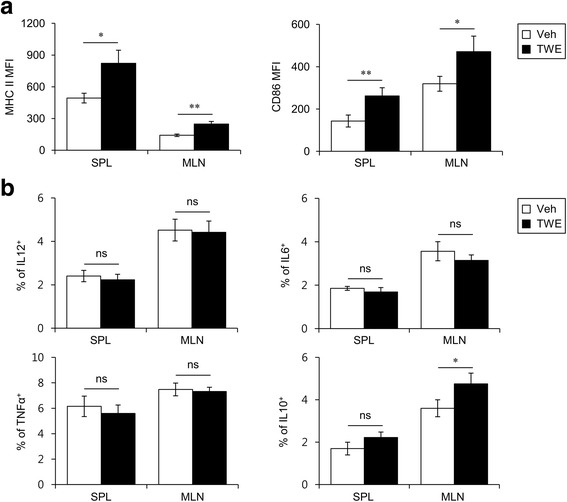
TWE induces an increase in maturation and production of anti-inflammatory cytokine but does not alter pro-inflammatory cytokine production by DCs. **a** Either TWE or vehicle was supplemented in the drinking water of B6 mice for 5 days (approximately 2 mg TWE/day). Subsequently, the surface expressions of MHC II and CD86 molecules on the DCs from the spleen and MLN were assessed by flow cytometry. The graphs represent the mean fluorescence intensities (MFI). The mean values ± SD (*n* = 5, * *P* < 0.05, ** *P* < 0.01) are shown. **b** Either TWE or vehicle was supplemented in the drinking water of B6 mice. After 5 days of TWE ingestion, the frequencies of IL12p40, IL6, TNFα, or IL10-producing DCs of the spleen and MLN were assessed by flow cytometry. The mean values ± SD (n = 5, * *P* < 0.05) are shown

TWE is known to have anti-inflammatory properties because TWE can suppress the production of inflammatory mediators such as prostaglandin E2 and nitric oxide (NO) [8, 9]. However, the effects of TWE on innate immune cells such as DCs have not been clearly defined. Thus, we decided to investigate whether in vivo treatment with TWE might affect the maturation of DCs. To test this possibility, we chose to deliver TWE orally into mice because taheebo is typically ingested as a tea. B6 mice were fed with 2 mg TWE per day in their drinking water for 5 days. Subsequently, the expression of MHC II and CD86, which are markers for DC maturation, was examined using splenic DCs isolated from mice fed with TWE-containing water. We found that TWE induced the up-regulation of both MHC II and CD86 in DCs, which indicated that TWE possesses some adjuvant abilities (Fig. 1a). However, TWE had little influence on the production of pro-inflammatory cytokines, including IL12, IL6, and TNFα, in both splenic and MLN DCs. Notably, TWE could induce an increase in IL10 secretion in MLN DCs, but not in splenic DCs (Fig. 1b). These results indicate that the oral administration of TWE enhanced the maturation of DCs but did not alter pro-inflammatory cytokine production in DCs.

### DCs are dispensable for helper T cell polarization by in vivo TWE treatment

**Fig. 2:**
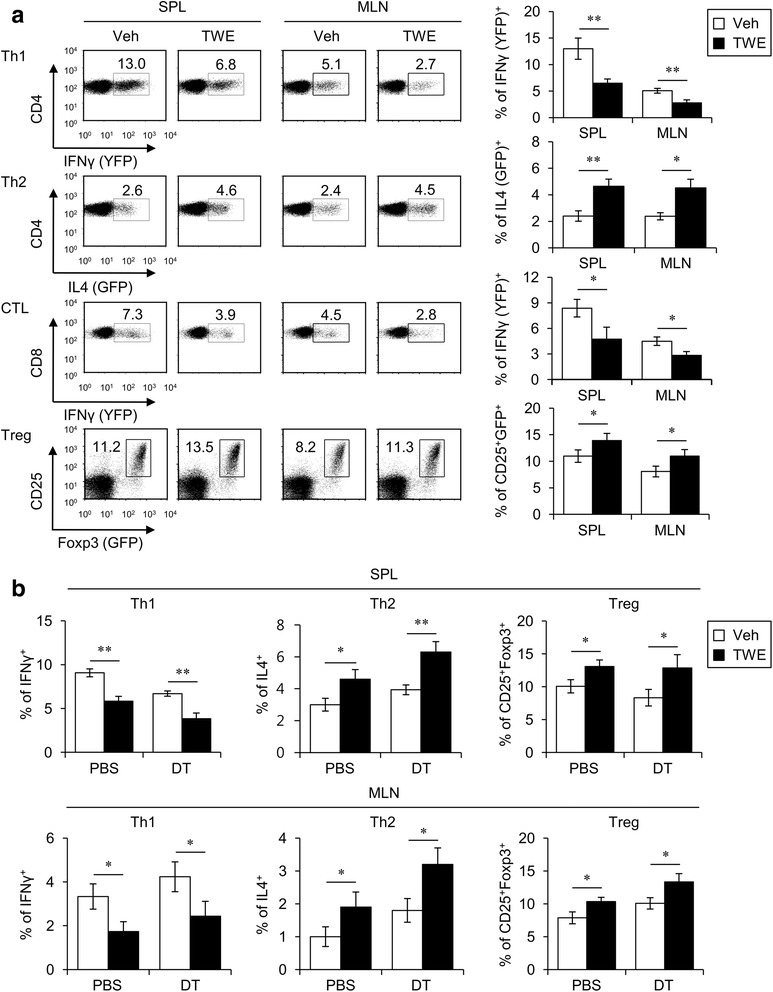
DCs are dispensable for TWE-mediated T cell polarization. **a** Either TWE or vehicle was supplemented in the drinking water of yeti (IFNγ/YFP), 4get (IL4/GFP), and Foxp3 (GFP) reporter mice for 5 days. Subsequently, the level of reporter protein expression was assessed in CD4^+^ T cells (CD3^+^CD4^+^), CTLs (CD3^+^CD8^+^), and Treg cells (CD3^+^CD4^+^CD25^+^) using flow cytometry. The mean values ± SD (*n* = 4, * *P* < 0.05, ** *P* < 0.01) are shown. **b** Either TWE or vehicle was supplemented in the drinking water of DT (120 ng/mouse)-treated CD11c-DTR tg mice. Three days later, DT was intraperitoneally reinjected for the further depletion of DCs. Two days after the second DT injection, the mice were sacrificed and subsequently single cells were prepared from the spleen and MLN. Intracellular IFNγ, IL4, and Foxp3 productions were assessed in CD4^+^ T cells (CD3^+^CD4^+^) and Treg cells (CD3^+^CD4^+^CD25^+^) by flow cytometry. The data are representative of two independent experiments (n = 4, * *P* < 0.05, ** *P* < 0.01)

To investigate whether TWE could regulate the adaptive immune system, we evaluated the profile of CD4^+^ T cell subsets in TWE-treated mice. TWE treatment markedly reduced the frequency of IFNγ-producing CD4^+^ and CD8^+^ T cells but increased the frequency of IL4-producing or Foxp3-expressing CD4^+^ T cells compared with the control mice (Fig. 2a). Because DCs are the most efficient initiator of the adaptive immune response, we next assessed whether the TWE-mediated polarization of T helper cells is dependent on DCs. To test this idea, we took advantage of CD11c-specific DTR tg mice, in which CD11c^+^ DCs can be depleted by a single intraperitoneal injection of DT. DT-induced depletion of DCs could not alter the differentiation of CD4^+^ T cells despite TWE treatment, which indicated that DCs are dispensable for TWE-mediated T helper cell differentiation (Fig. 2b). Collectively, these data suggest that antigen-presenting cells (APCs) other than DCs (e.g., macrophages and B cells) might be sufficient for the induction of T helper cell differentiation by in vivo TWE treatment.

### TWE enhances both maturation and M2 polarization of macrophages

**Fig. 3:**
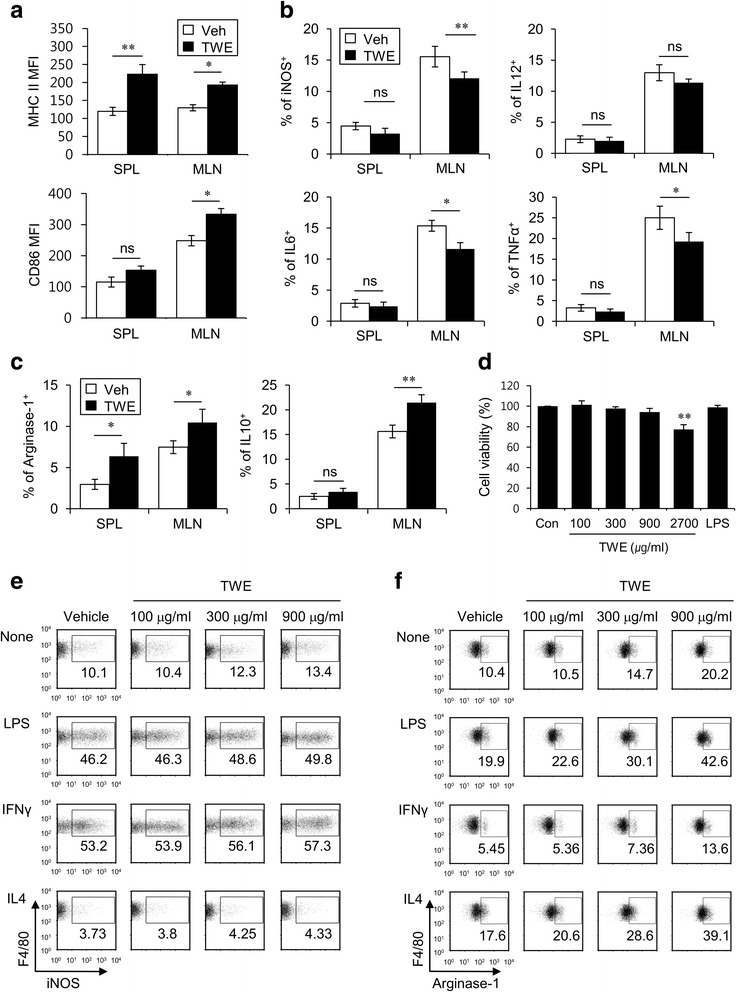
TWE enhances maturation and M2 polarization of macrophages. **a-c** Either TWE or vehicle was supplemented in the drinking water of B6 mice for 5 days (approximately 2 mg TWE/day). Subsequently, the surface expression of MHC II and CD86 molecules (**a**) and intracellular IL12p40, IL6, TNFα, iNOS (M1 markers) (**b**), arginase-1, and IL10 (M2 marker) (**c**) secretions were assessed by flow cytometry in macrophages (CD11c^−^CD11b^+^F4/80^+^) from the spleen and MLN. The mean values ± SD (*n* = 5, * *P* < 0.05, ** *P* < 0.01) are shown. **d** RAW264.7 cells (1 × 10^6^ cells) were stimulated with TWE (100, 300, 900, and 2700 μg/ml), LPS (1 μg/ml), or vehicle for 12 h. The cell viability (%) was expressed as 7-AAD negative percentage relative to the untreated control cells (*n* = 3, ** *P* < 0.01). **e, f** RAW264.7 cells (1 × 10^6^ cells) were stimulated with TWE (100, 300, and 900 μg/ml) or vehicle in the absence or presence of LPS (1 μg/ml), IFNγ (100 ng/ml), or IL4 (20 ng/ml) for 12 h. The cells were collected, and their productions of iNOS (**e**) and arginase-1 (**f**) were determined by flow cytometry

Because emerging evidence indicates that macrophages are involved in regulating various immune responses, such as experimental autoimmune encephalomyelitis (EAE), we next investigated whether TWE regulates both maturation and phenotypes of macrophages. To this end, we isolated mononuclear cells from the spleen and MLN of mice at day 5 after vehicle or TWE treatment. First, we found that TWE treatment induced a significant increase of MHC II molecules in macrophages from the spleen and MLN. However, TWE-mediated up-regulation of CD86 expression was only seen in macrophages from the MLN but not from the spleen (Fig. 3a). Second, the production of iNOS (M1 marker) and pro-inflammatory cytokines, including IL12, IL6, and TNFα, in macrophages was significantly decreased in the MLN but not in the spleen following TWE treatment (Fig. 3b), whereas the production of arginase-1 (M2 marker) and the anti-inflammatory cytokine IL10 in macrophages was significantly increased in the MLN by TWE treatment (Fig. 3c). These findings suggest that TWE treatment induces macrophages to polarize towards the M2 phenotype in the MLN.

To directly confirm whether TWE treatment was able to change the phenotype of macrophages by affecting macrophage polarization, we examined the in vitro TWE-mediated polarization of macrophages. To this end, first of all, the effect of TWE on cell viability was evaluated using RAW264.7 macrophage cell line and 7-AAD method. Compared to controls, cell viability was not significantly decreased in TWE concentrations ranging from 100 μg/ml to 900 μg/ml, which were thus used in the next experiments (Fig. 3d). Upon in vitro stimulation of macrophages with TWE, we found that TWE treatment had little effect on iNOS expression in LPS- or IFNγ-primed macrophages. Because IL4 is known to promote macrophages toward the M2 type, however, IL4-primed macrophages exhibited a decreased expression of iNOS upon in vitro TWE stimulation (Fig. 3e). In contrast, in vitro TWE stimulation significantly enhanced arginase-1 expression (M2 marker) in both unprimed and LPS-primed macrophages. Furthermore, to some extent, TWE could induce macrophages that were polarized to the M1 phenotype by IFNγ to increase arginase-1 expression. Under the M2-inducing IL4 priming condition, TWE treatment significantly increased the expression of arginase-1 in macrophages in a dose-dependent manner (Fig. 3f). Taken together, these results indicate that TWE modulates macrophage maturation and polarization towards the anti-inflammatory cytokine-producing M2 macrophage phenotype.

### Oral administration of TWE protects mice from DSS-induced colitis

**Fig. 4:**
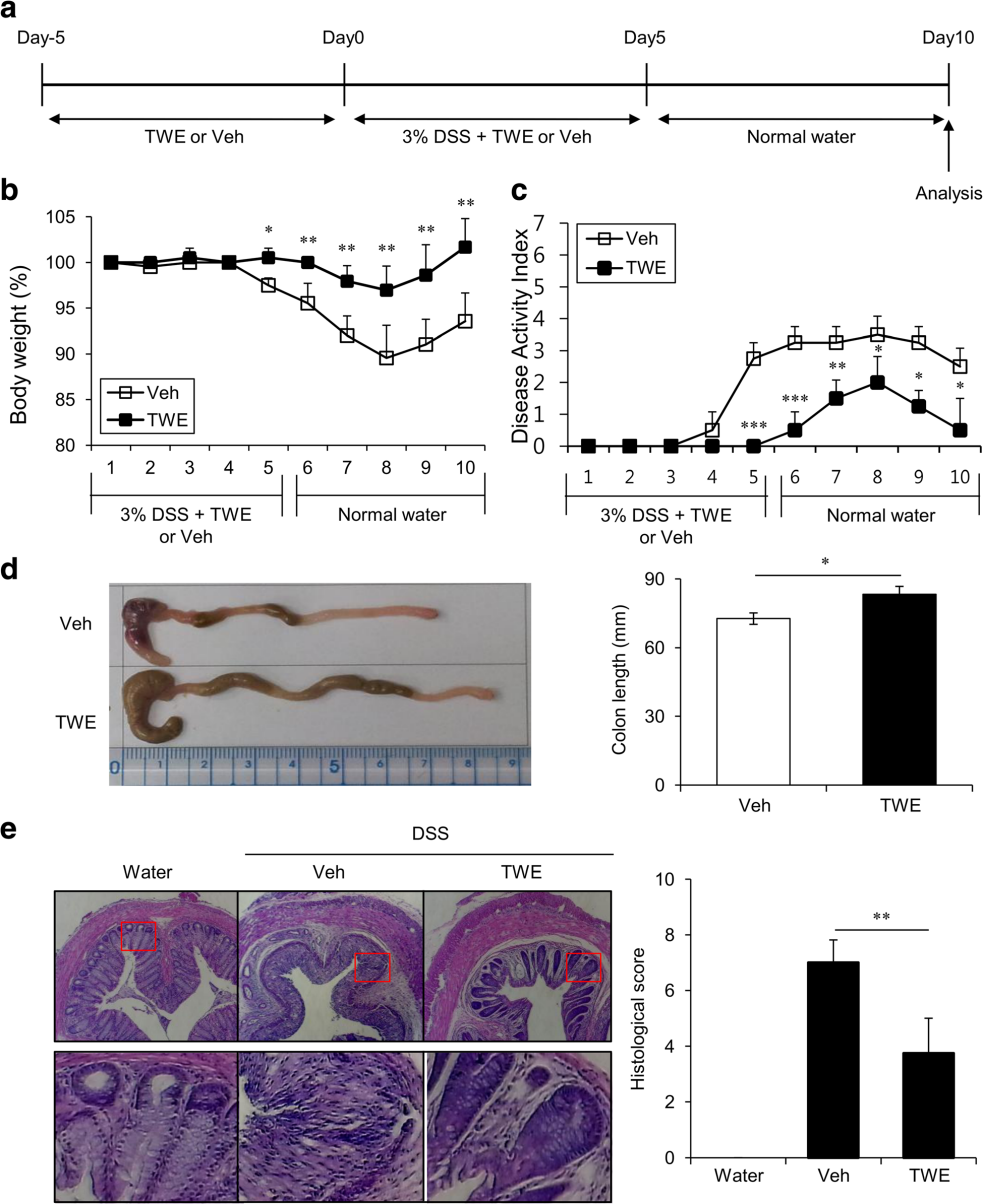
TWE protects mice from DSS-induced colitis. **a** Experimental scheme for TWE treatment in the DSS-induced colitis model. **b** Body weight change, shown as a percentage change relative to the body weight at day 0, was measured daily after DSS-induced colitis with or without TWE treatment. The mean values ± SD (*n* = 5, * *P* < 0.05, ** *P* < 0.01, *** *P* < 0.001) are shown. **c** Weight loss, stool consistency, and fecal blood were scored to provide the DAI for each group. **d** On day 10, the colons were removed, and their lengths were measured. The photos of representative colons are shown. The mean values ± SD (n = 5, * *P* < 0.05) are shown. **e** Histological examination of the colon tissue was performed at day 10 (left panel). The histological score was measured for colon cellular infiltration and tissue disruption (right panel). Representative colon tissue sections are shown. The mean values ± SD (n = 5, ** *P* < 0.01) are shown

The pathogenesis of IBD is characterized by the excessive expansion of pro-inflammatory Th1 and Th17 cells and by the simultaneous reduction of Treg cells [1]. Because TWE has potent anti-inflammatory effects on innate and adaptive immunity, we investigated whether the oral administration of TWE could modulate the development of experimental colitis in mice. To test this hypothesis, we adopted a method for DSS-induced colitis, as described in the Methods section. Briefly, the mice were fed with either TWE in their drinking water or normal water without TWE as a negative control for 5 days (approximately 2 mg TWE per day). These mice were subsequently fed with either 3% DSS plus TWE or 3% DSS without TWE in their drinking water for 5 days to induce acute colitis. After DSS treatment, the two groups of mice were fed with DSS-free normal water for five more days before sacrifice for analysis (Fig. 4a). We found that the body weight of the TWE-untreated mice was decreased from day 4 to day 7 post-DSS treatment and then slowly returned to the normal level through day 10, whereas the body weight loss of TWE-fed mice was noticeably attenuated (Fig. 4b). Furthermore, the TWE-treated group of mice had much lower levels of DAI (i.e., weak diarrhea and less bleeding in the feces) than did the controls (Fig. 4c). In addition, we found that the TWE-treated mice displayed a much less shortened length of the colon than did the control mice during DSS-induced colitis (Fig. 4d). From histological sections of colonic tissues, we found that TWE-treated mice displayed less severe signs of colonic inflammation (i.e., loss of epithelial crypts, mucosal edema, and infiltration of inflammatory cells) compared with the vehicle group (Fig. 4e). Taken together, these results demonstrated that the oral administration of TWE could prevent mice from DSS-induced colitis.

### TWE treatment attenuates the inflammatory immune response in experimental colitis

**Fig. 5:**
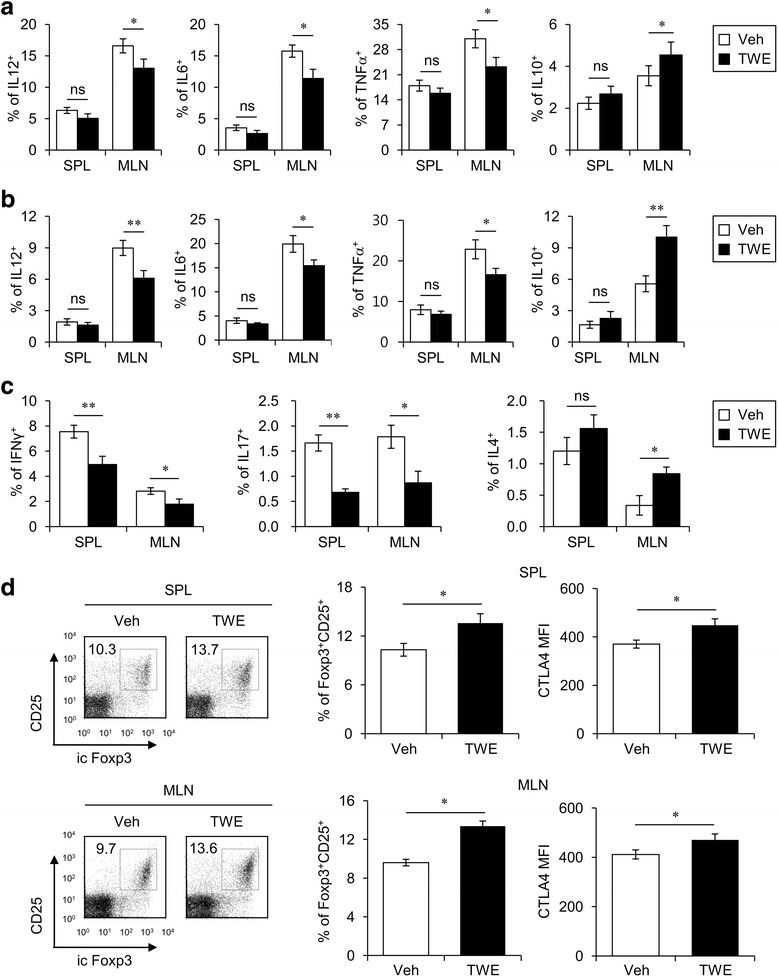
TWE attenuates inflammatory responses in experimental colitis. **a-d** Mononuclear cells were collected 10 days after 3% DSS treatment in the presence or absence of TWE. **a, b** Intracellular IL12p40, IL6, TNFα, and IL10 productions were measured in the DCs (**a**) and macrophages (**b**) from the spleen and MLN using flow cytometry. The mean values ± SD (n = 5, * *P* < 0.05, ** *P* < 0.01) are shown. **c** Intracellular IFNγ, IL17, and IL4 productions were measured in the CD4^+^ T cells (CD3^+^CD4^+^) from the spleen and MLN using flow cytometry. The mean values ± SD (n = 5, * *P* < 0.05, ** *P* < 0.01) are shown. **d** Intracellular Foxp3 expression was measured in the Treg cells (CD3^+^CD4^+^CD25^+^) from the spleen and MLN using flow cytometry. In addition, the expression level of CTLA4 in Treg cells from the spleen and MLN (CD4^+^CD25^+^Foxp3^+^) was analyzed by flow cytometry. The graphs represent the MFI. The mean values ± SD (n = 5, * *P* < 0.05) are shown

We next investigated the immunological mechanisms by which TWE treatment inhibited DSS-induced colitis in mice. Mucosal immune responses are counter-balanced through the interaction between pro- and anti-inflammatory cytokines. For instance, intestinal inflammation is mediated by IL12-induced Th1 cells and IL6-induced Th17 cells [1, 2]. In contrast, the anti-inflammatory cytokine IL10 is known to contribute to mucosal homeostasis in the intestinal epithelium [13]. Thus, we examined the cytokine profile following TWE treatment during DSS-induced colitis. To this end, we measured the production of IL12, IL6, TNFα, and IL10 in DCs and macrophages at day 10 post-DSS treatment. The oral administration of TWE significantly decreased the levels of pro-inflammatory cytokines (IL12, IL6, and TNFα) but increased the anti-inflammatory cytokine IL10 in DCs and macrophages from the MLN (Fig. 5a and b). Upon TWE stimulation during DSS-induced colitis, the frequencies of both Th1 and Th17 cells were significantly diminished in both the spleen and MLN, whereas IL4-producing Th2 cells were remarkably increased in the MLN (Fig. 5c). Additionally, the frequency and expression level of the suppressive molecule CTLA4 on Treg cells were significantly increased by TWE treatment in both the spleen and MLN (Fig. 5d). These findings clearly indicate that TWE treatment induces the differentiation of anti-inflammatory T helper cells, such as Th2 and Treg cells, ultimately leading to the overall inhibition of inflammatory responses induced by DSS-induced colitis.

## Discussion

As public interest in natural products as a source of potential candidates for medicine is increasing, complementary and alternative medicines that use scientifically verified natural products have rapidly grown over the last decades in both the Eastern and Western world [14]. Bark products from the taheebo tree have been one of the natural products used as a traditional folk remedy for various diseases such as arthritis. Although many people drink taheebo as a tea, TWE has been noted as a source of natural products that can provide a health benefit beyond basic nutrition. Although the in vitro and in vivo anti-inflammatory effects of taheebo have previously reported [8, 9], the effects of TWE on intestinal immune responses have been poorly understood. Herein, we demonstrate that the oral administration of TWE led to the induction of Th2 and Treg differentiation via alterations in APCs, such as M2 polarization, and resulted in the inhibition of Th1 cells and IFNγ production by CD8^+^ T cells in the MLN. Furthermore, by employing the DSS-induced murine model of colitis, we clearly showed that TWE treatment was capable of attenuating the clinical symptoms of colitis in mice, which suggests that TWE could be an excellent reagent as natural products to improve the clinical symptoms of IBD.

Adaptive immune responses are controlled by innate immune cells such as DCs. Depending on the types of cytokines they secrete, DCs can influence the differentiation of CD4^+^ T cells that play critical roles in adaptive immunity. Tolerogenic DCs produce immunosuppressive IL10 rather than IL12 and express high levels of costimulatory molecules, such as MHC II and B7, when induced by thymic stromal lymphopoietin (TSLP). Because it has been shown that tolerogenic DCs can prime Th2 and Treg cells and also contribute to preventing excessive inflammation in the mucosal environment [15], we inferred that TWE could affect the differentiation of CD4^+^ T cells towards the Th2 and Treg cells via a DC-mediated pathway. However, our results showed that Th2 and Treg cells could be still induced upon TWE treatment, even in the case of DC depletion. Although DCs are the most potent APCs at initiating adaptive immune responses, it could be possible that other professional APCs such as macrophages are responsible for the modulation of T helper cells by TWE treatment. For example, it has been reported that lamina propria macrophages inhibit Th1 and Th17 differentiation but induce Treg differentiation during the intestinal immune response [3, 16]. Furthermore, gut-resident macrophages play a central role in oral tolerance and the expansion of Treg cells during the intestinal immune response to orally administered food antigen, which implies that macrophages act as local APCs in the intestine [17]. Based on the above reports, macrophages rather than DCs might be involved in tolerance in the intestine. Conversely, one recent study has reported that both DCs and macrophages are essential for optimal Th1 polarization during IBD-like pathogenesis in mouse infection models [18], which implies that DCs and macrophages participate synergically to induce Th2 and Treg polarization upon TWE treatment.

Foxp3^+^ Treg cells are known to play a critical role in maintaining tolerance against self-antigens. Recently, some studies have reported the reciprocal relationship between the Treg cell number and the severity of mucosal inflammation. For example, a decreased number of Treg cells was correlated with active IBD in patients [19], whereas an increased number of Treg cells inhibited the severity of an experimental colitis model [20, 21]. Thus, our finding that TWE treatment can induce Treg cells can explain how the oral administration of TWE could prevent DSS-induced colitis in mice. In addition, a couple of studies have demonstrated that Th2 polarization using helminths can be an effective way to control mucosal inflammation [22, 23]. Such alternative therapeutic approaches to induce the mucosal Th2 immune response have been shown to suppress the Th1 response, consequently protecting against colitis in mice. Therefore, the expansion of Th2 and Treg cells by TWE treatment might be one of the key mechanisms that contribute to inhibiting DSS-induced colitis.

Our results suggest that macrophages are among the key players for the TWE-mediated inhibition of colitis. Classically activated M1 (stimulated by IFNγ) and alternatively activated M2 (stimulated by IL4) are two major subsets of macrophage in the intestine [5]. Recently, it has been reported that the up-regulation of M2 macrophages suppressed the severity of colitis via IL10 receptor signaling [13] but that the down-regulation of M2 macrophages increased the susceptibility to colitis [24, 25]. In addition, M2 macrophages have been shown to be involved in Th2 polarization, and the consequent reciprocal IL4 production by Th2 cells can affect the phenotypic alteration of macrophages from M1 to M2 [26]. Because M2 macrophages were increased in response to TWE stimulation, our findings suggest that the mutual interaction between M2 and Th2 cells might account for the suppressive activities of TWE in colitis. Emerging evidence supports the idea that the interaction between gut microbiota and intestinal immunity plays a central role in maintaining intestinal homeostasis [2]. Intriguingly, Jang et al. showed that *Lactobacillus spp.* belonging to the Firmicutes phylum ameliorated colitis in mice by polarizing M1 macrophages to the M2 phenotype [27, 28]. Because one previous study demonstrated that the major component of TWE helped to maintain lactic acid-producing beneficial bacteria, such as *Lactobacillus spp.* and *Bifidobacterium spp.,* but preferentially inhibited the growth of harmful bacteria such as *Clostridia* and *E. coli* [29], we cannot exclude the possibility that the inhibition of DSS-induced colitis by TWE treatment could be attributed to the distinct effects of TWE on gut microbiota. Further investigations need to be done to address this issue.

The TWE used in this study was prepared by extracting the water-soluble portions from taheebo barks immersed in 4 °C cold water for 24 h, as described in the Methods section. Thus, TWE is not a pure compound and instead consists of several active components, including naphthoquinone, β-lapachol, and polyphenols, which all have anti-inflammatory activities. For example, naphthoquinone induced the decreased production of NO (M1 phenotype) in macrophages stimulated with LPS [30] and promoted Th2 immune responses (M2 inducer) in conditions of antigen-related lung inflammation [31, 32]. β-lapachol displayed anti-inflammatory effects in EAE [33] and polyphenols had potent anti-inflammatory activities [34, 35]. Therefore, it will be of interest to examine which components of TWE are primarily responsible for its anti-inflammatory activity in colitis.

Considering our results, we might anticipate a couple of concerns about TWE-mediated Th2-deviation. First, there is one type of colitis called UC that is known to be mediated by Th2-dominant immune responses. Unlike Th1-mediated CD, Th2-mediated UC actually became worse after helminth infection [36]. If TWE prevents colitis via a Th2-polarized manner, it could worsen UC upon TWE treatment. However, although it has been reported that UC pathogenesis is dependent on IL13 production by natural killer T (NKT) cells, we did not find that TWE treatment activated NKT cells to produce cytokines such as IL13 (data not shown). This finding implies that TWE may not have much influence in the pathogenesis of UC, though it should be investigated to confirm whether TWE has any side effects on UC in the future. Second, TWE treatment might increase the induction of Th2-mediated allergic responses, such as atopic dermatitis (AD) and asthma. Recently, very interesting studies have reported on the correlation between allergy and mucosal immune responses. Feeding food allergens with Th2-skewing adjuvants alone did not promote allergic responses in the skin [37] and acute diarrhea in the intestine [38] without repeated prior skin sensitization. In addition, intestinal allergic responses were suppressed by orally ingested food allergen before skin sensitization occurred [37]. Thus, we speculate that orally administered TWE has little influence on causing skin allergic diseases such as AD. In support of our speculation, we found that the oral administration of TWE did not affect the severity of AD in NC/Nga mice (unpublished preliminary data).

## Conclusions

The present study suggests the following scenario whereby TWE can modulate DSS-induced colitis. First, the oral administration of TWE initiates the phenotypic alteration of innate immune cells such as DCs and macrophages, which results in the polarization of macrophages towards the M2 phenotype as well as the tolerization of DCs. Second, such anti-inflammatory changes in the innate immune system promotes CD4^+^ T cells to differentiate into Th2 and Treg cells but restrains the development of Th1 and Th17 cells in the intestine. Consequently, TWE treatment could effectively prevent acute DSS-induced murine colitis. Taken together, our findings suggest that TWE will be an excellent natural product to help ameliorate the symptoms of various inflammatory immune disorders such as IBD. Very recently, Choi et al. reported that the intake of TWE could reduce the development of high-fat diet-induced obesity [39]. Because obesity is closely linked to macrophage activation, our present findings might provide experimental evidence that TWE treatment can help maintain the homeostasis of metabolic diseases, including obesity and diabetes, by switching macrophage polarization from M1 to M2 phenotype. It will be very interesting to investigate whether this is the case.

## Abbreviations

APCs: antigen-presenting cells
B6: C57BL/6
CD: Crohn’s disease
CTLA4: cytotoxic T lymphocyte-associated protein 4
DAI: disease activity index
DCs: dendritic cells
DSS: dextran sulfate sodium
DT: diphtheria toxin
DTR: diphtheria toxin receptor
EAE: experimental autoimmune encephalomyelitis
Foxp3: forkhead box P3
IBDs: inflammatory bowel diseases
IFNγ: interferon γ
IL: interleukin
iNOS: inducible nitric oxide synthase
LPS: lipopolysaccharide
MACS: magnetic activated cell sorting
MLN: mesenteric lymph node
NKT: natural killer T
RA: retinoic acid
tg: transgenic
Th: T helper type
TNFα: tumor necrosis factor α
Treg: regulatory T
TWE: taheebo water extract
UC: ulcerative colitis
